# Conceptualising global health: theoretical issues and their relevance for teaching

**DOI:** 10.1186/1744-8603-8-36

**Published:** 2012-11-14

**Authors:** Mike Rowson, Chris Willott, Rob Hughes, Arti Maini, Sophie Martin, J Jaime Miranda, Vicki Pollit, Abi Smith, Rae Wake, John S Yudkin

**Affiliations:** 1 UCL Institute for Global Health, 30 Guilford Street, London, WC1N 1EH, UK; 2 UK Department for International Development, 1 Palace Street, London, SW1E 5HE, UK; 3 General Practitioner and Medical Educationalist, NHS Ealing, London, UK; 4 Independent consultant, London, UK; 5 CRONICAS Centro de Excelencia en Enfermedades Crónicas and Department of Medicine, School of Medicine, Universidad Peruana Cayetano Heredia, Lima, Peru; 6 National Clinical Guideline Centre, Royal College of Physicians, London, UK; 7 Academic Clinical Fellow in Obstetrics and Gynaecology. Women's Health, Southmead Hosptial, Westbury on Trym, Bristol, BS10 5NB, UK; 8 Division of Global Health and Human Rights, Massachusetts General Hospital, Boston, MA, USA; 9 Juba Teaching Hospital, Juba, Sudan; 10 University College London, Gower Street, London, UK

**Keywords:** Global health, Medical education, Undergraduate, Curriculum, Globalization, Equity

## Abstract

**Background:**

There has long been debate around the definition of the field of education, research and practice known as global health. In this article we step back from attempts at definition and instead ask what current definitions tell us about the evolution of the field, identifying gaps and points of debate and using these to inform discussions of how global health might be taught.

**Discussion:**

What we now know as global health has its roots in the late 19^th^ century, in the largely colonial, biomedical pursuit of ‘international health’. The twentieth century saw a change in emphasis of the field towards a much broader conceptualisation of global health, encompassing broader social determinants of health and a truly global focus. The disciplinary focus has broadened greatly to include economics, anthropology and political science, among others. There have been a number of attempts to define the new field of global health. We suggest there are three central areas of contention: what the object of knowledge of global health is, the types of knowledge to be used and around the purpose of knowledge in the field of global health. We draw a number of conclusions from this discussion. First, that definitions should pay attention to differences as well as commonalities in different parts of the world, and that the definitions of global health themselves depend to some extent on the position of the definer. Second, global health’s core strength lies in its interdisciplinary character, in particular the incorporation of approaches from outside biomedicine. This approach recognises that political, social and economic factors are central causes of ill health. Last, we argue that definition should avoid inclusion of values. In particular we argue that equity, a key element of many definitions of global health, is a value-laden concept and carries with it significant ideological baggage. As such, its widespread inclusion in the definitions of global health is inappropriate as it suggests that only people sharing these values may be seen as ‘doing’ global health. Nevertheless, discussion of values should be a key part of global health education.

**Summary:**

Our discussions lead us to emphasise the importance of an approach to teaching global health that is flexible, interdisciplinary and acknowledges the different interpretations and values of those practising and teaching the field.

## Background

With increasing international investment in global health, coupled with growing academic and student interest, as well as the concern of activists and the general public, debates about what exactly defines ‘global health’ are widespread [1-4]. According to Koplan et al., ‘without an established definition [of global health] … we cannot possibly reach agreement about what we are trying to achieve, the approaches we must take, the skills that are needed and the ways that we should use resources’ [1] (p. 1993).

We welcome the renewed attention to debates around definition as this compels the academic community concerned with global health to consider further refinement of what the field should encompass. We have attempted our own definitions in the past^a^. In this article we choose to reflect on these definitions and ask what the surrounding debates tell us about how the discipline of global health has evolved so far, and where it might go in the future. The article then specifically relates these issues to the theory and practice of teaching global health at both undergraduate and postgraduate levels. An accompanying article [5] reviews trends in the teaching of global health in undergraduate medical curricula and uses this diagnosis of gaps to suggest areas where teaching might develop further.

The current article identifies a number of controversies raised by the debate about definitions that remain unresolved. The first set of issues concern the scope of the field, in terms of geographical coverage and the range of topics it should cover. The second set of issues concern the purpose of global health education. Should this be to teach students about the effectiveness of interventions and policies that aim to improve global health or also to promote discussion of the goals of global health themselves? This analysis reveals disagreement about the values that underpin the field and about how the theoretical or discursive world of global health should be connected with the ‘real’ world of global health policy and practice.

These debates seem to be especially marked where an area of academic study has close connections with a field of practice, and are not limited to global health. Development studies is the most relevant example, where similar disputes about naming, meaning, values and goals have been particularly sharp [6,7]. We will draw on these discussions as we proceed.

## Discussion

### Naming ’global health’

The origins of recent debates about the meaning of global health can be traced through the history of public health and medicine. Co-operation between nations on health issues dates back at least to the attempts to control the epidemics of disease such as plague that swept Europe during the Renaissance. However, the field that came to be described as ‘international health’ had its roots in the period of colonisation, war and increasing migration and trade at the end of the nineteenth century. Intensified international health activity sought to guard the people of Europe and North America against cross-border infections and to protect their colonial interests [8].

The twentieth century saw the gradual establishment of the major institutions of international health, culminating in 1948 with the foundation of the World Health Organization (WHO). The accent of WHO’s work in its first 30 years was on combating infectious disease, notably through its global and country-level campaigns towards eradication of smallpox and malaria. In the same vein, ‘international health’ work at leading academic centres of public health in the developed world – where they were concerned with overseas health issues – was also largely focussed on disease control.

Changes which were to reshape both understanding and practice of international health came from two directions in the 1970s. Firstly, in developed countries there was a shift away from the focus on individual prevention to an approach that recognised the need to build ‘healthy public policy’. Policy documents – notably the famous 1974 Lalonde report in Canada [9] – began to promote the idea that a person’s health was the result of many different influences, including genes, behaviour, the social environment and health services [10]. This legitimated a focus on these broader determinants of health in policy and research, both nationally and internationally. Simultaneously the WHO, inspired by its charismatic Director-General Dr. Halfdan Mahler, formulated the Alma-Ata Declaration on Primary Health Care in 1978 [11]. With its focus on the underlying causes of ill-heath, its recommendations for inter-sectoral action and its promotion of widespread political and economic change, the Declaration was both of its time politically, and ahead of it in its radical recommendations for the re-organisation of public health and health services.

These developments helped to shift understanding of the scope of international health from its historic emphasis on tropical disease and cross-border infection control. During the 1980s and 1990s, the emerging interest in health economics in the wake of welfare state crises in the rich nations, and the application of this discipline to the health services of developing countries, also contributed to a further broadening of the field’s perspective. These decades also saw the growth of vertical programmes to tackle key diseases, spurred in part by the emerging HIV/AIDS epidemic. This development encouraged greater discussion amongst global health practitioners on ways to prevent vertical programmes from having a detrimental effect on the clamour for comprehensive primary health care that arose at Alma-Ata [12].

Since the 1990s, parts of public health academia, as well as campaigners and concerned policymakers, have continued to engage with the social factors that constrain the ability of individuals to maintain healthy lifestyles. The focus on the regulation of the marketing practices of the tobacco and food industries by national and international health bodies are two notable examples of this. The report of the recent WHO Commission on Social Determinants of Health has strengthened this emphasis on the ‘upstream’ factors that determine health worldwide [13]. As a result, academics, policymakers and practitioners from all kinds of disciplinary backgrounds have made contributions to health debates.

This broadening of perspective has been further encouraged by deepening globalization, leading to some academics and practitioners proposing a new field of ‘global health’, a process that took place alongside WHO’s effort to reposition itself as leader in global health in the early 1990s [14]. The former head of health promotion at WHO, Ilona Kickbusch, has suggested that global health has three primary concerns: the global distribution of health and disease and their determinants; the impact of globalization on health; and the changing nature of global health governance [15]. Kickbusch differentiates these concerns from those of international health which, she argues, is more focussed on the ‘health problems in developing countries and the flow of resources and knowledge from the developed to the developing world’. Similarly, Lee and Collin argue that a global health issue is one where 'determinants circumvent, undermine or are oblivious to the territorial boundaries of states and, thus, beyond the capacity of individual countries to address through domestic institutions’ [16] (p. 3). Koplan and colleagues echo this thinking in their recent contribution to the definition debate. Global health, they argue, is:

an area for study, research, and practice that places a priority on improving health and achieving equity in health for all people worldwide. Global health emphasises transnational health issues, determinants, and solutions; involves many disciplines within and beyond the health sciences and promotes interdisciplinary collaboration; and is a synthesis of population-based prevention with individual-level clinical care [1].

Note how in these definitions the new concept of global health is hitched to globalization, suggesting a focus on both international interdependence in health and how economic, environmental, political and social processes – on a worldwide scale – affect people's health. ‘Global health’ does not limit itself to the study of the developing world, and as such appears to avoid the ‘us’ and ‘them’ approach that has characterised much international health discourse. An explicit recognition of the relevance of other disciplines has also entered the definition, as has regard for equity and population-level health interventions.

But do these definitions lay to rest debates about definition? We would suggest that there are still debates around three aspects of the definition: firstly, the *object of knowledge* of global health in terms of geographical scope and coverage of issues; secondly, the *types of knowledge* required to practise the field and how far engagement with cross-disciplinary perspectives really goes; and finally, the *purpose of knowledge* – relating to debates about goals and values within the field of global health and, linked with this, the need to create space for discussion. Andy Sumner and Michael Tribe have posed similar questions about development studies that have informed the framework of analysis of this paper.

### Object of knowledge

Globalization, understood as a worldwide transformation whereby global processes and decisions increasingly influence national ones, has challenged the idea that international or global health can simply concern developing countries. For Koplan et al. [1] the term ‘global’ thus refers to any health issue that concerns many countries or is affected by transnational determinants such as climate change or urbanisation, or transnational solutions such as polio eradication, such that the ‘global in global health refers to the scope of the problems, not their location’ (p. 1994).

But what is a global health issue that concerns many countries? Koplan and colleagues run the full gamut from infectious and chronic disease to accidents: ‘global health has to embrace the full breadth of important health threats … burden of illness should be used as a criterion for global-health priority setting’ (p. 1994). But this might imply that issues lower down the burden of disease ranking or with lower ‘importance’ are not global health ones. And it focuses on the health conditions rather than the wide-ranging determinants of the health conditions, although the authors also acknowledge that these are worthy of study.

Bozorgmehr and colleagues have dissected in detail many of these issues around the object of knowledge, noting the difficulties in understanding the implications of the word ‘global’ [4]. They settle on an understanding of global as supra-territorial determinants ‘which impact on and thereby link the social determinants of health *anywhere* in the world; but not necessarily *everywhere* or to the same extent’ [4] (p. 5, emphasis in original). In their understanding the global is not a separate ‘transnational’ set of issues, but a description of a deeper reality wherein people’s health everywhere may or may not be influenced by global forces.

While the focus on linkages and commonalities across space may well be an important feature of global health we would also do well to remember differences. Firstly, the specificity of location or, more appropriately, *context*, is key to understanding any global health issue. The underlying emphasis on commonalities between nations in recent definitions of global health obscures the need to recognise differences between countries at different levels of development and with different social, political and economic systems, and the resulting need for context-specificity. Striking differences exist between countries across the world – both within and between those groups of countries commonly referred to as ‘developed’ and ‘developing’ – including the types of health problems that affect them, the nature of their welfare systems and economies, and the level of foreign involvement in economy, policy-making and society. The relationship between researchers and researched is also ‘far more problematic if those involved in the research are at a completely different level of economic and political power’ [17] (p. 10), and researchers face formidable challenges in some low- and middle-income countries in obtaining reliable, valid and representative data. Even if it is the case that, for example, poverty and gender discrimination exist in all countries, their characteristics and causes are often radically different and require specialised knowledge to analyse. These differences imply the need for significant amounts of attention and resources to be devoted to adaptation of tools and techniques used by researchers in poor countries, to capacity building of developing country institutions and to context-sensitive theory building.

Second, the focus on commonalities neglects the importance of positionality in determining teachers’, researchers’ and practitioners’ perspectives on global health or their sense of which global health issues are important. As a group comprising mainly British academics and doctors, who are therefore highly positionally specific, we are wary of being prescriptive about what to view as appropriate elements of the field of global health. It follows that there is no sense in creating defined lists of global health issues that are essential to the field. These will change over time and space, with the perspective of the observer.

Third, the focus on commonalities can of course be justified within medical education for teaching students about diseases which affect both ‘us’ (in richer countries) and ‘them’ (in poorer countries) (or which can be carried by ‘them’ to our countries). Given the levels of within country and international migration that have occurred in the last thirty years and the cosmopolitan nature of many of the urban environments in the rich world in which future medical students will serve, this interest in ‘common issues’ is not unreasonable. However, it may mean that global health research, practice and education priorities are influenced by the concerns of the richer rather than poorer countries. This echoes a long tradition in global health whereby large amounts of international aid have been poured into interventions and research on (infectious) diseases in poor countries that have the potential to affect populations in rich nations [18].

What principles can we draw from this discussion, and how do they inform a perspective on global health education? First, that the focus on commonalities is not wrong, but it clearly cannot be the only criterion for determining what the focus of global health education should be. Differences – sometimes radical ones – also have to be acknowledged. This is not a contradiction; it simply creates an interesting dilemma for programmes about where to place emphasis. Second, and related to this, an emphasis on ‘transnational issues’ in any definition may arbitrarily limit the scope of what is deemed to be a global health activity. It would be better instead to recognise that the object of knowledge of global health may shift depending on the position and interests of the actors involved; and that recognising this fact, actors should seek to strike a balance in their approaches to global health where possible – for example, by undertaking teaching that covers both similarities and diversities in global health. Indeed, we should welcome and cherish diversity in the teaching of global health in different contexts, and seek to learn from these different contexts about what is perceived to be of importance in global health.

### Types of knowledge

Global health is a field that is characterised by vast differences in the phenomena that can be studied, stretching from economic, political and social relationships to biological processes and even to the technologies that deliver health-sustaining resources such as water, sanitation and agricultural improvements^b^. All of these phenomena need to be studied in their context and with appropriate disciplinary input.

The recent debates about definition all recognise the need for cross-disciplinary approaches [1-5]. Out in the real world this is already occurring: we increasingly see anthropologists brought into global health programmes to evaluate why medical interventions are accepted or rejected by communities [19]; sociologists asked to explain why child mortality rates vary, where quantitative techniques give inconclusive answers [20]; labour market economists to analyse patterns of employment in the health professions within and across countries [21,22]; and mathematicians to understand the impact of interventions on patients or individuals working in complex and ever-changing health systems [23]. Within global health teaching the situation is similar, with a wide variety of different disciplines – biomedicine, social sciences and humanities – being taught on undergraduate and postgraduate global health courses.

Despite this the proposed definitions are rather vague about the extent of influence of disciplines outside public health. Indeed, Fried et al. [2] argue that global health *is* public health; and Koplan et al. [1] echo this by suggesting that while many disciplines need to be involved in global health, fundamentally what is needed is ‘a synthesis of population-based prevention with individual-level clinical care’. These authors are perhaps themselves working within the long-standing debate within medicine about the relative effort that should be devoted to prevention and cure as well as to social versus individual determinants of health. The broader perspectives of public health are clearly relevant for global health, but they should not be equated. As Beaglehole and Bonita [3] note, while ‘in many countries public health is equated primarily with population-wide interventions, global health is concerned with all strategies for health improvement, whether population-wide or individually based health care actions, and across all sectors, not just the health sector’ (pp. 1–2).

Partly the issue here is a sensitivity from those outside the public health and medical fields to the dominance of these perspectives in global health as it is practiced, researched and taught. But global health is not simply about health problems, as all the authors of recent definitions recognise (though the vast majority of these authors are themselves from biomedical or public health backgrounds): it is also about the underlying determinants of those problems, which are social, political and economic in nature. The involvement of lawyers, economists and engineers should be as unremarkable in the field of global health as those of doctors and other health care professionals. Yet most of the institutions of global health governance, research, education and practice are dominated by those trained as doctors. This inevitably adds a ‘biomedical’ slant to most global health practice, arguably leading to inappropriate interventions and repeated policy failure. We therefore welcome the conclusions of the *Lancet* Commission on the future of health professional education, which called for more interprofessional education [24]. We also welcome attempts to show, practically, how knowledge from different disciplines can be brought to bear on global health, as Kleinman has done [25].

There is another important reason why global health is not public health – or, to be more specific, why global health is not *only* public health – and it lies in the international contexts within which global health operates. Fried et al. [2] comment that ‘global health and public health represent a single field with a long tradition of bringing scientifically validated approaches, technologies, and systems to bear on the world's most pressing health needs’ (p. 537). The reason why this approach appears to us as flawed, and why it belongs to a different era of global health, lies in its understanding of global health as a technical process, unconnected to the political and social context within which it operates. It is therefore posited on a vision that sees those with the greatest ‘scientifically validated approaches, technologies, and systems’ as those who should lead the movement for global health. Implicitly, therefore, the argument is for a ‘Western’ scientific approach to global health problems, which is more in line with previous conceptions of ‘international health’.

We would approach things differently, and suggest that global health is about much more than interventions, but also that, as mentioned elsewhere in this paper, it must be context-specific and be flexible enough to respond to the differences in these contexts. Literature abounds on the mistakes made when global development is viewed solely as a technical concern [26-28]. In the 21^st^ century, it is vital that global health, as a field of research, practice and education, does not make the mistake of viewing technical interventions as magic bullets to improve the lives of people across the world. For this reason, the disciplines of sociology, economics, political science, anthropology and others must be brought to bear on global health, so that it becomes a field that challenges preconceived norms and expectations on the value of different types of knowledge, rather than reinforcing them.

The need for interdisciplinarity is as important in the teaching of global health in medical curricula as it is in research and practice: understanding the multitude of interrelated factors that underpin individual and population-level health outcomes is impossible without reference to disciplines outside of biomedicine and public health. It is encouraging that public health too is moving towards this more reflexive and broader approach.

### Purpose of knowledge

As the focus on technical prescriptions for health improvements suggests, global health is an instrumental field, so resembling development studies, another field characterised by cross-disciplinarity and global perspectives. In other words, it ‘seeks to make a difference’ [29]. As Sumner and Tribe indicate in the case of development studies, there are varying degrees of instrumentality, from weakly instrumental approaches that involve theoretical and abstract knowledge-seeking, to more applied, strongly instrumental forms.

All recent definitions of global health place a strong emphasis on instrumentality. Much of the argument is about how to ‘do’ global health better (for example by bringing in cross-disciplinary perspectives), and the emphasis is on reaching agreement on definition in order to improve global health. Yet global health is not just about understanding *how* to intervene but also *why* intervention takes place and the power relations that characterise the contemporary world. This is a reflexive, as opposed to instrumental form of knowledge, and space should be made, alongside analysis of policy and practice, for what Kothari [7] (p. 7) calls ‘critical and radical academic investigation of ideas and histories.’ This is a vital aspect of contemporary debates on global health.

This becomes clear when we move to discussion of the values that underlie any instrumental approach to global health. Koplan et al. [1] and Fried et al. [2] argue that a key tenet of any definition of global health is its focus on making a difference to inequities in health. Fried et al. contend that the discipline of public health is strongly characterised by ‘dedication to better health for all, with particularly attention to the needs of the most vulnerable populations, and basic commitment to health as a human right’. Kerry et al. similarly argue that ‘medical education training programmes must engage the explosively growing interest in global health with a primary goal to reduce the global burden of disease…’ [30] (p. 5). It is also clear that much writing about global health – particularly in the medical field – is characterised by a similar concern for equity, ‘attention to the social determinants of health’ or at the very least ‘health improvement’.

There is, however, a question over whether values should be included in definitions. Definitions of academic disciplines tend to remain ideologically neutral (and rather ‘technical’ in nature), and inclusion of the term equity compromises this goal. Economics can be defined as ‘the study of the production, distribution and consumption of wealth in human society’ [31], while political science is the study of ‘politics at all levels of which the most basic is the study of conflict’ [32]. The definition of economics does not focus on the acquisition of capital, nor does political science emphasise any particular political system. Global health should follow this lead, because to stray from this approach would imply particular political solutions to global health problems. In particular, the term equity carries with it an implicit endorsement of progressive solutions to global health problems. This is apparent in the view of Koplan et al. [1], which holds equity to be coterminous with ‘reduction of health disparities.’ Most academics would argue that this goal is likely to be achieved only through a reordering of society in favour of the least well-off. The ideological neutrality of a definition of global health would therefore be compromised through this definition.

The second issue we have with the inclusion of equity lies in the definition of the term. Equity – or its synonym, fairness – has different meanings across the world, and not just with regard to health policy. Mackintosh in her work on social settlements argues that health systems across the world come to embody levels of inequality that members of that society perceive to be justifiable or, to use a different term, equitable. Some level of inequality is viewed in many societies as justifiable, and the factors that influence this view vary markedly from society to society:

‘The patterns of inequality in any society are framed by strong legitimizing conventions of thought: from caste-based social distinctions carrying religious significance, via deeply embedded assumptions of gender inequality, to shared expectations that the more educated should receive higher incomes’ [33] (p. 182). 

It is not controversial to suggest, therefore, that equity – fairness – has different meanings in different contexts. One only has to look at the relatively homogenous western world to realise that the term has significantly different meanings in, say, Sweden, the United Kingdom and the United States. In order for equity to be included in any definition of global health, therefore, it must be defined by those using it. As fairness means different things to different people, and to different societies, this appears impossible. It is particularly problematic to argue that equity should be part of any definition of global health when the people making the definitions form such a narrow group: academics working in global health.

Third, the fact that many of those teaching, researching and practising global health across the world – including the authors of this paper – are committed to global health equity and reduction of global health disparities, does not mean that those who do not share these values are not teaching or practising global health: to suggest so would be absurd. A commitment to equity (whatever that may mean) is not a prerequisite for involvement in the field, nor should it be.

We therefore agree with Bozorgmehr [4] (p. 14) that ‘definitions should abstain from attaching normative objectives a priori and factually describe what the field *is*, not what it ideally *should* be’ [italics in original]. Instead, again like Bozorgmehr, we find it preferable to argue that concepts and goals such as equity should be recognised as a key focus of debate within the field, not a central part of the definition, and that any ‘intervention’ or ‘solution’ to a problem always generates complex trade-offs for society. It is better for definitions to remain agnostic on values, whilst making it a key principle that space should be made to debate values, goals, concepts and choices in educational (and all other) global health contexts.

Instrumental approaches to global health are also behind the increasing concern with defining core competencies and learning outcomes in global health teaching [34-36]. This is valuable as there is a danger that ‘without well thought-out competencies and educational approaches, doctors and medical students may lack the foundation to participate in international global health programs’ [34] (p. 5), resulting in ‘harm to patients and their communities’ [35] (p. 2). Nevertheless the drive to competency should be combined with reflexivity about fundamental values and established ways of doing things, lest education in this area descend into rote-learning of lists of prescribed subjects, which would be inimical to the spirit of debate that should characterise global health education, and which clearly energises students.

### Summary

We have argued that the evolving debates about definition are a useful prompt about how to teach global health. In the light of this we suggest a number of principles to inform and guide educators in their decisions.

First, we – like many of those cited above – take seriously the historical thrust of changes in the definition of international health to global health, as well as the underlying motors of those changes. This involves a commitment to explaining and understanding the underlying causes of ill-health and to understanding the commonalities that underlie people’s health around the world. However, we would argue that doing this involves going substantially beyond the traditional focus of public health on simply identifying underlying causes, to developing the tools needed to understand these determinants as they manifest themselves across time and space. It thus necessitates a multi-disciplinary approach and a context-driven one. It means emphasising differences and making space for consideration of these differences when defining curricula.

Second, and related to the first issue, it is important not be too prescriptive about what falls under the rubric of ‘global health’: the key point is that the meaning of global health – the object of knowledge – will shift depending on the position of the actors studying or otherwise engaged with it. This helps us avoid taking a ‘Western,’ or a biomedical, approach to prescribing the content of curricula. ‘Our’ concerns may not be the same as ‘theirs’. Where possible, this needs to shine through in curricula.

Third, there is a need to recognise that values are a key part of any discussion of global health and students should be encouraged to engage with this. Definitions have a tendency towards technical prescription, even when the definitions themselves mention values like equity. Instead, we advocate putting all values ‘up for grabs’ in discussion and helping students to take a critical approach. While it is true that the practice of global health is often about intervening to alleviate illness and disadvantage, reflection on these interventions needs encouraging by creating a space in global health curricula for investigation of the ideas, assumptions and values that underpin them. A focus on the acquisition of competencies in specific areas of global health should balance a focus on the acquisition of a competency in critical thinking.

### Endnotes

^a^Cited in Bozorgmehr, 2010.

^b^Sumner A and Tribe M 2004 note the same breadth in development studies.

## Abbreviation

WHO: World Health Organization.

## Competing interests

The authors declare that they have no competing interests.

## Authors’ contributions

MR led the development of the framework for the paper, wrote the first draft of the paper and co-ordinated the final rewrite of the paper. CW contributed to redrafting of the paper and co-ordinated the final rewrite of the paper. RH commented on initial drafts, helped develop the framework and contributed to the final rewrite of the paper. AM commented on initial drafts and contributed to the final rewrite of the paper. SM commented on initial drafts and contributed to the final rewrite of the paper. JJM commented on initial drafts and contributed to the final rewrite of the paper. VP commented on initial drafts and contributed to the final rewrite of the paper. AS commented on initial drafts and contributed to the final rewrite of the paper. RW commented on initial drafts and contributed to the final rewrite of the paper. JSY commented on initial drafts and contributed to the final rewrite of the paper. All authors read and approved the final manuscript.

Note: the findings, interpretations and conclusions expressed in this article are those of the authors and do not necessarily reflect the views of their employers.

## Authors’ information

MR: MSc, Senior Teaching Fellow, UCL Institute for Global Health, 30 Guilford Street, London WC1N 1EH, UK.

CW: PhD, Senior Teaching Fellow, UCL Institute for Global Health, 30 Guilford Street, London WC1N 1EH, UK.

RH: MB ChB MPH BSc, Health/Nutrition Advisor, UK Department for International Development, 1 Palace Street, London SW1E 5HE, UK.

AM: MBBS BSc MRCGP DFSRH MSc Med Ed, General Practitioner and Medical Educationalist NHS Ealing, London, UK.

SM: MSc, Independent consultant, London, UK.

JJM: MD, PhD, CRONICAS Centro de Excelencia en Enfermedades Crónicas and Department of Medicine, School of Medicine, Universidad Peruana Cayetano Heredia, Lima, Peru.

VP: BSc, MSc, Health Economist, National Clinical Guideline Centre, Royal College of Physicians, London, UK.

AS: MBCHB BSc, Obstetrics and Gynaecology Trainee. Great Western Hospitals NHS Foundation Trust, Marlborough Road, Swindon, SN3 6BB, UK.

RW: BM BSc DTM&H, Fellow, Division of Global Health and Human Rights, Massachusetts General Hospital, Boston, MA, Juba Teaching Hospital, Juba, South Sudan.

JSY: FRCP, Emeritus Professor of Medicine, University College London, Gower Street, London WC1E 6BT, UK.
